# Mr. Hamilton, on the Gravel

**Published:** 1810-10

**Authors:** W. Hamilton

**Affiliations:** Ipswich


### *To the Editors of the Medical and Physical Journal.*

Sir,

HAVING perused many valuable and rare cases in your useful Journal, I take the liberty to transmit for your consideration one which does not seem altogether void of interest.

R. Smith, ætat. 63, had been subject to frequent attacks of the gravel. He was seized in the month of May last with pain, extending across his back and loins; much difficulty in passing urine, which was turbid, and precipitated a considerable quantity of thick sediment on standing for a few hours.

He was ordered laxative and mucilaginous medicines, which, in the course of a few weeks, so far relieved him that he left off medicine, and continued pretty well till the beginning of July, when he again applied. He could now scarce pass any urine without the greatest difficulty, screaming out violently when he made the attempt. He also complained of a pain continually, extending along the course of the urethra, which was so tender that he could scarcely bear the least pressure applied to it. At this time neither the penis nor scrotum appeared the least inflamed or swelled.

Bougies and the catheter were often attempted to be introduced, but neither could be passed into the bladder. The stricture appeared near the prostate. On withdrawing the catheter, near an inch of its point was covered with bloody mucus. Its introduction was attended with such excruciating pain, that he could seldom be prevailed on to suffer the attempt;—and as he voided his urine in a full stream, and in considerable quantity, although accompanied with the most violent pain imaginable, the neighbours comparing it to the throes of labour; the attempt to pass it was therefore laid aside. After the urine was voided, the pain continued for a few minutes, and then subsiding left the sensation of

*scalding* and *smarting*, as he expressed it. This was caused, no doubt, by the abradation of the inner surface of the urethra. He continued in a similar state for near a fortnight, when the membranous part of the urethra was observed, in the course of one night, to have swelled to near three times its natural size. This remained stationary for some days, when it began to increase rapidly, distending all the base of the scrotum, especially the left side. Its substance, which had continued till now quite relaxed, became also suddenly distended and anasarcous. At this time he did not pass more than two ounces of water in the course of the twenty-four hours. A rupture of the urethra or bladder therefore seemed evident: the size of the scrotum became now immense, and the pain less severe.

Warm fomentations had been constantly applied to the pubis and hypogastric region, which gave him more ease than opium, conium miculatum, or other narcotics. He was now harassed with frequent reachings and vomitings, and had no appetite, to which diarrhœa and singultus soon succeeded, and his dissolution appeared fast advancing.

As he would not submit to any operation, and for temporary relief, the scrotum was punctured in several places; and water exuded in considerable quantity from this time till his decease, which happened about thirty-six hours after.

### Examination of the Parts.

About six hours after I obtained, with some difficulty, permission to inspect the diseased parts. The scrotum was so much distended that it covered all the circumjacent parts, not only concealing the perinæum, but extending over the anus, when placed on his back. On applying partial pressure to its surface, the impression remained, for some time, as in other anasarca.

An incision was made on the left of the septum scroti, and after passing through near an inch in depth of cellular substance, the scalpel plunged at once into a sac, in withdrawing which, purulent matter, instead of urine, rushed out as from a divided artery. The puncture was enlarged, and in less than two minutes, more than a *quart* was collected of this apparently well-digested pus. On examining the urethra, I found that it was not only ruptured from the neck of the bladder to within about three inches of the glans penis, but that corpus spongiosum had, in a great measure, formed the cyst in which the matter was contained; the rupture of which into the scrotum caused, no doubt, its sudden distention, tention, which is noticed above. The neck of the bladder was also destroyed, having two or three perforations into it, through which I could easily pass my fingers. I then felt whether there were any calculi, but found none; on the contrary, the bladder itself was contracted to less than half the natural size, and consequently much thickened: and on its left side, near the entrance, was found a hard schirrhous tumor about the size of a pigeon's egg, but no abscess. The testes were in separate cavities from that in which the matter was contained, and appeared healthy.

You will excuse this rough sketch, and be assured of my regret, that prejudice should not have suffered a more minute detail.

I remain, Sir,
with great respect, yours, &c.
W. HAMILTON.

*Ipswich, August* 2, 1810.

